# Resveratrol increases resistance of mouse oocytes to postovulatory aging *in vivo*

**DOI:** 10.18632/aging.101494

**Published:** 2018-07-23

**Authors:** Qiu-Xia Liang, Yi-Hua Lin, Chun-Hui Zhang, Hong-Mei Sun, Liang Zhou, Heide Schatten, Qing-Yuan Sun, Wei-Ping Qian

**Affiliations:** 1 Department of Reproductive Medicine, Peking University Shenzhen Hospital, Shenzhen, Guangdong 518036, China; 2 State Key Laboratory of Stem Cell and Reproductive Biology, Institute of Zoology, Chinese Academy of Sciences, Beijing 100101, China; 3 Department of Veterinary Pathobiology, University of Missouri, Columbia, MO 65211, USA; 4 University of Chinese Academy of Sciences, Beijing 100101, China; * Equal contribution

**Keywords:** resveratrol, postovulatory aging, oocyte, oxidative stress

## Abstract

After ovulation, metaphase II oocytes undergo a time-dependent deterioration *in vivo* or *in vitro*, which is referred to as postovulatory oocyte aging, a process during which a series of deleterious molecular and cellular changes occur. In this study, we found that short-term injection of resveratrol (3,5,4'-trihydroxystilbene) effectively ameliorated oxidative stress-induced damage in postovulatory oocyte aging of middle-aged mice *in vivo*. Resveratrol induced changes that delayed the aging-induced oocyte deterioration including the elevated expression of the anti-aging molecule Sirtuin 1 (SIRT1); it reduced intracellular reactive oxygen species (ROS) level, and improved mitochondria function. In addition, these beneficial changes may also help to prevent apoptosis. Taken together, our data suggest that resveratrol can effectively protect against postovulatory oocyte aging *in vivo* primarily by preventing ROS production.

## Introduction

Successful pregnancy requires high-quality embryos with embryonic developmental potential that is largely dependent on oocyte quality, considering that the oocyte provides all the necessary factors for orchestrating key events during early embryo development [1]. Oocyte deterioration as a result of postovulatory aging negatively impacts embryonic development [2–4]. Postovulatory aging refers to a degenerative process in MII oocytes which is strongly associated with fertilization failures within a period of time after ovulation. Postovulatory aging occurs both *in vivo* and *in vitro*. In humans and certain primates, owing to no visible signs of ovulation, fertilization may occur between an *in vivo* aged oocyte and freshly ejaculated spermatozoa. Oocytes employed in assisted reproductive technologies (ART) are often inevitably subjected to postovulatory aging. In light of these considerations, it is highly important to understand the mechanisms underlying the oocyte degeneration process, which may lead to solutions to preventing or delaying postovulatory aging and allow successful ART and embryo engineering procedures.

Postovulatory aging is highly associated with reduced fertilization rates [5], poor embryo quality [2–4] and fetus development [2,6,7]; however, the molecular mechanisms that control this process still remain poorly understood. Accumulating evidence indicates that antioxidant enzymes including Mn-superoxide dismutase (Mn-SOD), catalase, and glutathione peroxidase reduce ROS, whereas the production of ROS rises gradually with increasing maternal age [8]. Oxidative stress is strongly linked to the declined oocyte quality [9,10]. In addition, because the mitochondrial DNA (mtDNA) is not protected by histones, aging oocytes are very vulnerable to oxidative stress damage and mtDNA mutations. Dysfunctional mitochondria affect the oocyte’s metabolic capacity, and thus impair adenosine triphosphate (ATP) production. The ATP content in oocytes is correlated with embryonic development and implantation [11]. Elevation of intracellular ROS also inactivates nicotinamide adenine dinucleotide (NAD+)-dependent histone deacetylase SIRT1 [12], and decreased expression of SIRT1 accelerates postovulatory aging of oocytes *in vitro* [13]. In addition, high level of oxidative stress is considered an inducer of apoptosis of aging oocytes [14,15].

Resveratrol is a type of natural phenolic compound and a phytoalexin produced by several plants in response to injures or attacks by pathogens. It exhibits therapeutic effects against various diseases including cancer [16–18], diabetes [19–21], obesity [22,23], cardiovascular diseases [24,25], neurodegenerative disorders [26,27] and aging [28–31], and the effects are thought to be correlated to its anti-oxidative activity of resveratrol. In addition, it has been reported that resveratrol improved *in vitro* maturation of oocytes and enhanced the oocytes’ resistance to chemical reagents [32,33], heat stress [34] and cryopreservation-induced damage [35] in various species. Furthermore, resveratrol improves fertilization outcome of pig oocytes [36] and subsequent embryonic developmental potential [37,38]. Extensive studies about the effects of resveratrol on oocyte maturation are carried out *in vitro*, while *in vivo* research is rarely reported. In this study, we investigated the effects of resveratrol on delaying postovulatory aging of mouse oocytes *in vivo*.

## RESULTS

### Resveratrol maintains the survival of aging MII oocytes *in vivo*

**Figure 1:**
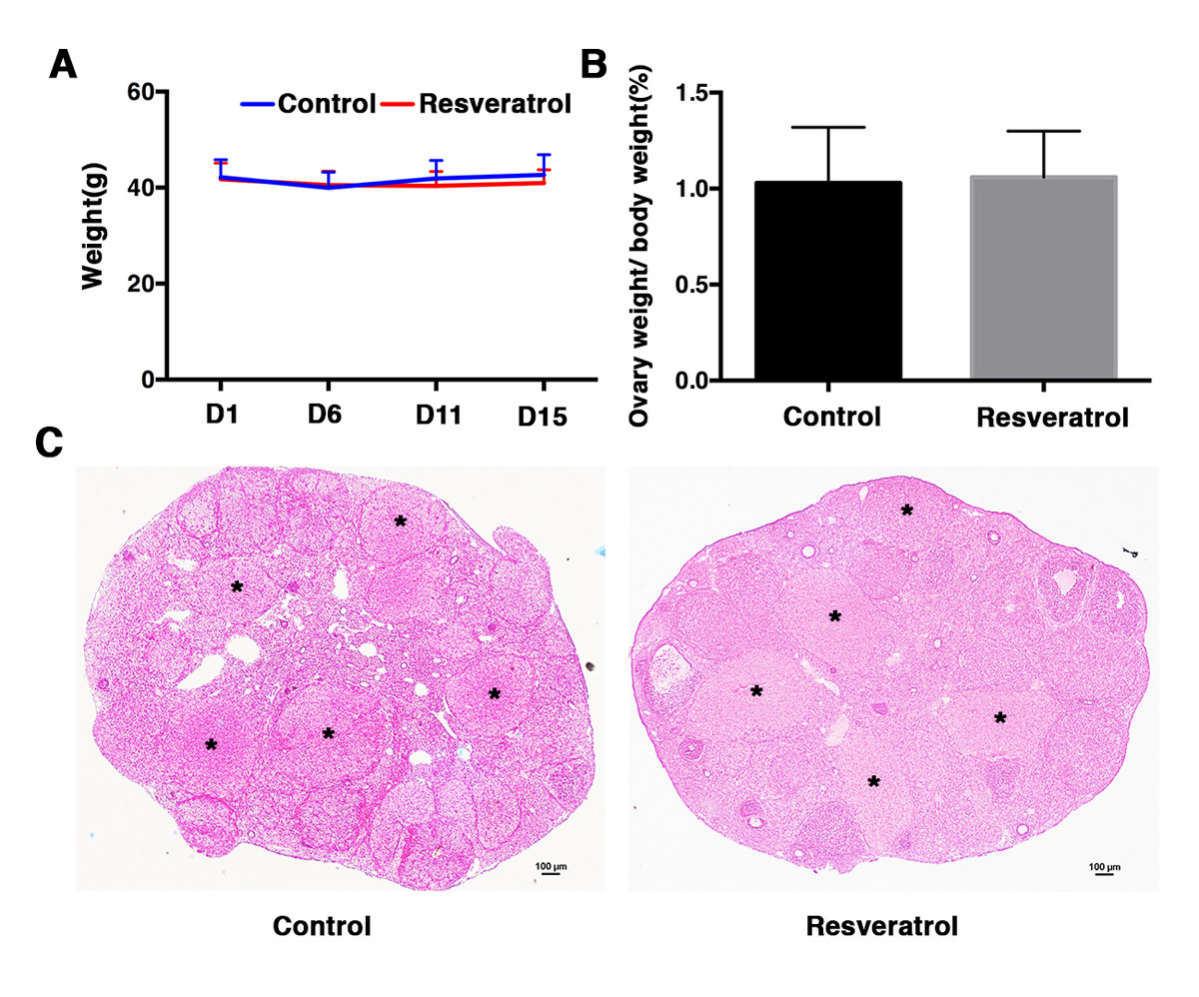
(**A**) Ovary weight of control and resveratrol-treated group at day 1, 6, 11 and 15 after treatment. For each time point, at least 30 mice of each group were used for analysis. Data are expressed as mean ± SEM of at least 6 independent experiments. (**B**) Ovary weight to body weight ratio of control and resveratrol-treated group at day 15 after treatment. At least 30 mice of each group were used for analysis. Data are expressed as mean ± SEM of at least 6 independent experiments. (**C**) Representative ovarian histology of control and resveratrol-treated group: 48 h after PMSG injection, hCG was administrated and ovaries were collected 24 h later for histological analysis. Black asterisks indicate corpus luteum. Scale bar: 100 μm.

**Figure 2:**
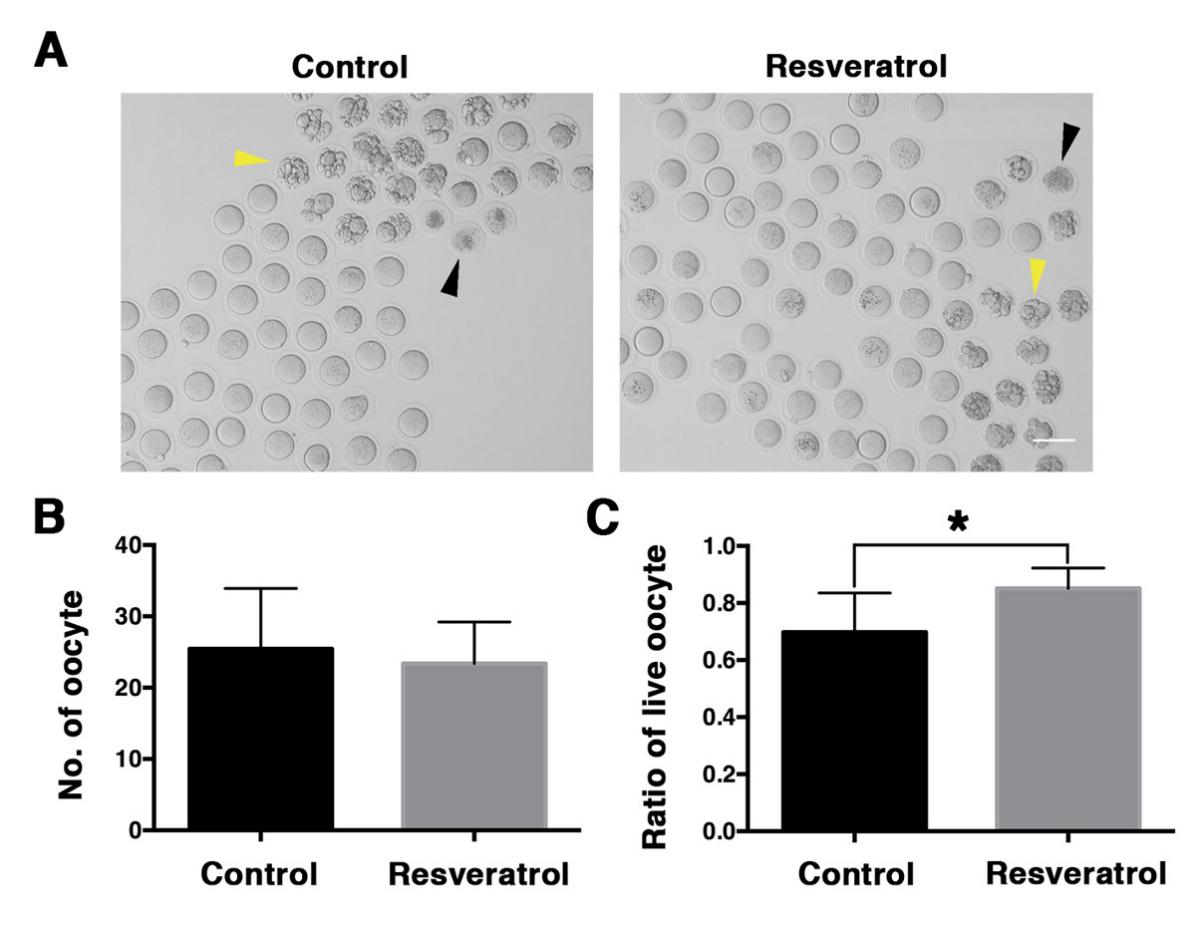
**Morphological evaluation of aging MII oocytes derived from control and resveratrol-treated mice *in vivo*.** (**A**) Microscopy images of aging MII oocytes from control and resveratrol-treated mice *in vivo*. Yellow and black arrowheads indicate apoptotic and death oocytes, respectively. (**B**) The number of aging MII oocytes from control and resveratrol-treated groups. Data are expressed as mean ± SEM of at least 5 independent experiments. (**C**) The ratio of live oocytes in control and resveratrol-treated groups. Data are expressed as mean ± SEM of at least 5 independent experiments. *Significantly different (P < 0.05).

To investigate the potential role of resveratrol in postovulatory oocyte aging *in vivo*, female ICR mice at 7-8 months of age (from the time at which fertility begins to decline) were injected with resveratrol (50 mg/kg BW/day) for 15 consecutive days. Forty-eight hours after PMSG injection, hCG was administrated, and 24 h later postovulatory *in vivo* aging oocytes and ovaries were collected. Firstly, the weights of control and resveratrol-treated mice were monitored on D1, D6, D11 and D15, and the body weight of resveratrol-treated mice was slightly lighter than controls from D11 to D15, but without significant differences (41.78 ± 3.34 vs. 42.19 ± 3.67 g; 40.5 ± 2.93 vs. 39.95 ± 3.26 g; 40.37 ± 3.01 vs. 41.91 ± 3.76 g; 40.97 ± 2.78 vs. 42.68 ± 4.21 g, n= 30, P > 0.05) (Figure 1A). No difference was observed in ovarian weight ratio between the resveratrol-treated group and the control group (1.06 ± 0.24% vs. 1.03 ± 0.29%, n= 30, P > 0.05) (Figure 1B). The histological assessment of ovarian sections showed that the corpus luteum formed normally in both resveratrol-treated and control groups (Figure 1C), which revealed the normal occurrence of ovulation. A portion of the aging oocytes showed apoptosis or death in both groups (Figure 2A). We counted the total number of super-ovulated oocytes, and no obvious variation was found in the resveratrol-treated group and the control group (23.39 ± 5.82 vs. 25.44 ± 8.48, n= 30, P > 0.05) (Figure 2B). We calculated the rate of living oocytes, and found that it was significantly higher in the treated group compared to the control group (85.11 ± 7.15% vs. 69.79 ± 13.75%, n= 30, P < 0.05) (Figure 2C).

### Resveratrol elevates the expression of SIRT1

**Figure 3:**
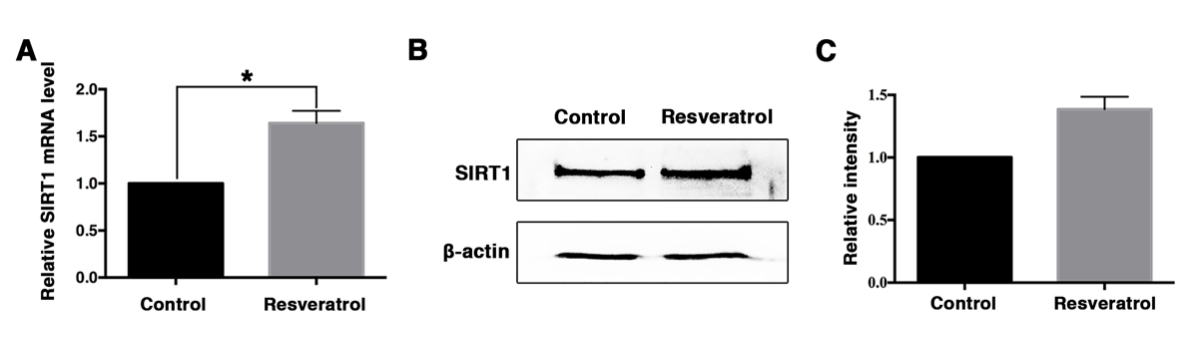
**The expression of SIRT1 in aging MII oocytes derived from control and resveratrol-treated mice *in vivo*.** (**A**) The expression of SIRT1 mRNA detected by quantitative RT-PCR. Error bar denotes SEM of three experiments. (**B**) Western blot detection of SIRT protein levels in aging MII oocytes from control and resveratrol-treated mice. (**C**) Quantitative analysis of gray intensity in control and resveratrol-treated groups.

Previous studies reported that SIRT1 could protect mouse oocytes from postovulatory aging and resveratrol was an activator of SIRT1. Accordingly, we analyzed the mRNA and protein level of SIRT1 in the oocytes of control and resveratrol-treated mice. The results of real-time PCR revealed that at mRNA level SIRT1 was significantly elevated in aging oocytes from resveratrol-treated mice, when compared to the control group (Figure 3A). Besides, immunoblotting analysis showed that the level of SIRT1 protein was also higher in aging oocytes of resveratrol-treated mice compared to the control group (Figure 3B and 3C).

### Resveratrol reduces ROS levels in aging MII oocytes *in vivo*

**Figure 4:**
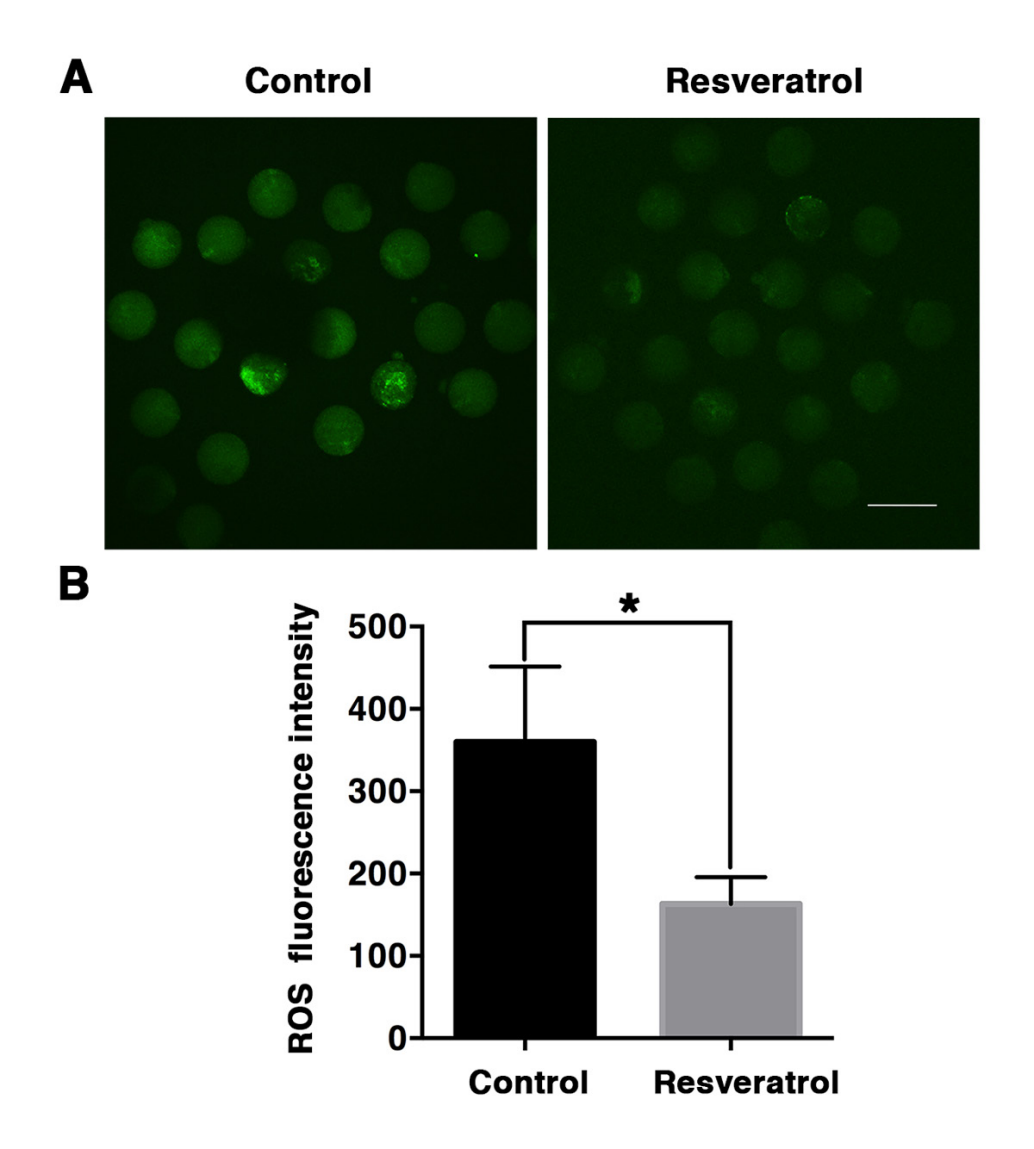
**Detection of ROS production in MII oocytes during aging *in vivo*.** (**A**) Representative confocal images of DCF fluorescence in control and resveratrol-treated oocytes. ROS production was measured by fluorescent probe DCFA-DA (green). Scale bar: 100 μm. (**B**) The relative levels of intracellular ROS determined by quantitative fluorescence intensity. Data are expressed as mean ± SEM of at least 5 independent experiments. *Significantly different (P < 0.05).

Towards determining whether resveratrol might protect against oxidative stress, aging MII oocytes derived from control and resveratrol-treated mice were collected to measure the level of intracellular ROS. The results showed that ROS in aging MII oocytes from control groups displayed clustered distributions; by comparison, ROS distribution in aging MII oocytes from resveratrol-treated groups was relatively uniform (Figure 4A). Moreover, the ROS fluorescence intensity of oocytes from resveratrol-treated mice was significantly lower compared to control aging oocytes (163.23 ± 32.56 vs. 359.88 ± 91.71, n= 70, P < 0.05) (Figure 4B), indicating a reduced production of ROS.

### The mtDNA copy number is lower in resveratrol-treated oocytes, but mitochondrial distribution is not affected

**Figure 5:**
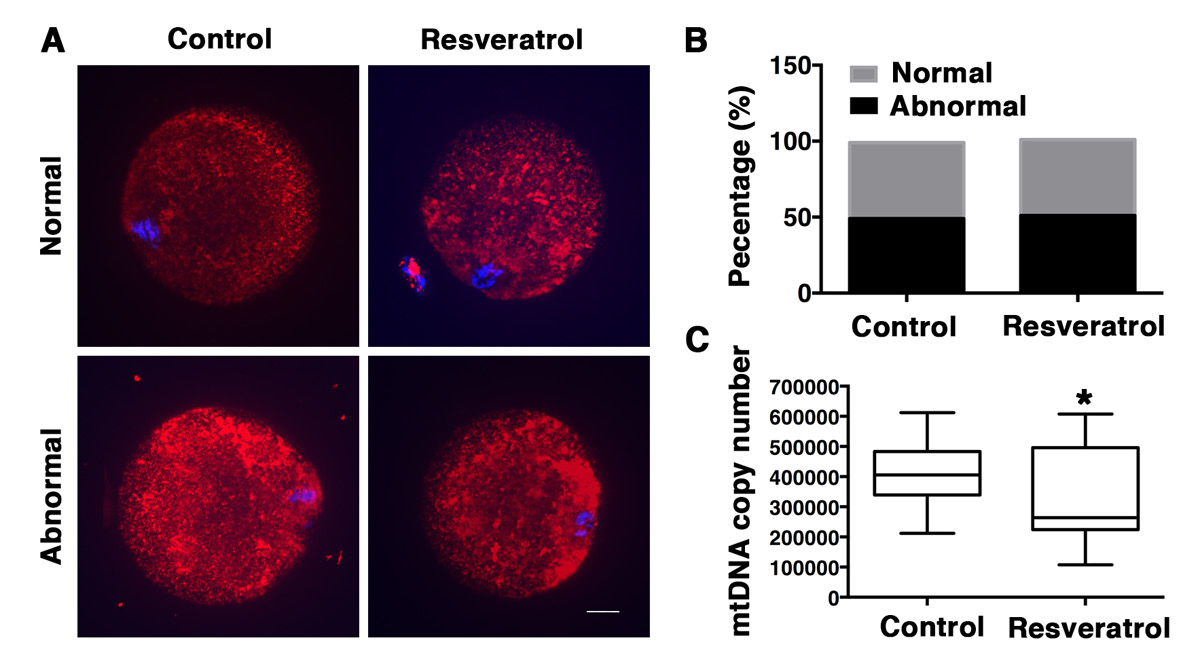
**Analysis of mitochondrial distribution and mtDNA copy number in MII oocytes during aging *in vivo*.** (**A**) Confocal microscopy images of normal and abnormal mitochondrial distribution patterns in oocytes recovered from control and resveratrol-treated mice. Mitochondrial distribution patterns were detected using Mito Tracker Red. DNA was counterstained with Hoechst (blue). Scale bar: 10 μm. (**B**) Percentages of the normal and abnormal distribution patterns in control or resveratrol-treated oocytes, respectively. (**C**) The average copy number of mitochondrial DNA in control and resveratrol-treated oocytes. Data are expressed as mean ± SEM of at least 3 independent experiments. *Significantly different (P < 0.05).

Because damage to mitochondria is a known cause of increased production of ROS and mitochondrial dysfunction is presumably linked to postovulatory oocyte aging, we next examined mitochondrial distribution and copy number in aging MII oocytes from control and resveratrol-treated mice. The results indicated that mitochondria in MII oocytes from both groups displayed two distribution patterns: homogeneous distribution pattern and clustered distribution pattern (Figure 5A). The proportion of the two patterns was similar in the resveratrol-treated group and the control group (50, 50%, n= 40 vs. 49, 51%, n= 50, P > 0.05) (Figure 5B). In addition, real-time PCR was performed to count mtDNA copy numbers in the two groups. Unexpectedly, we found that the mtDNA copy number in the resveratrol-treated group was significantly lower compared with the control non-treated aging group (322,504 ± 27,979, n=27, vs. 396,841 ± 15,478, n=40, P < 0.05) (Figure 5C).

### Resveratrol does not improve spindle assembly and chromosome alignment in aging oocytes

**Figure 6:**
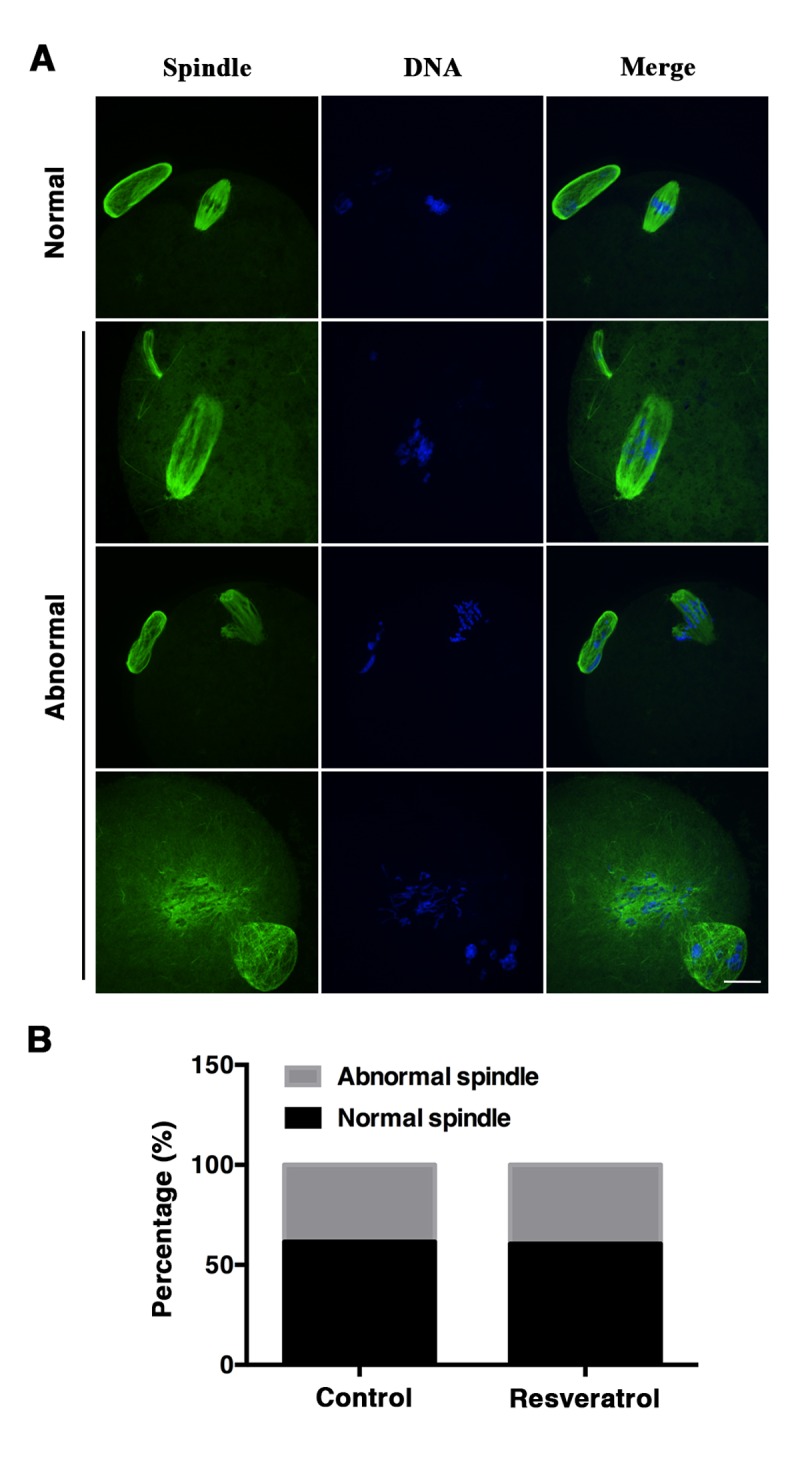
(**A**) Immunofluorescent images of normal and abnormal spindle morphology of control and resveratrol-treated oocytes. Oocytes were stained with α-tubulin antibody (green) and Hoechst (blue) to show spindle morphology and chromosome alignment, respectively. Scale bar: 20 μm. (**B**) Proportions of the normal and abnormal spindle morphology of aging MII oocytes from control and resveratrol-treated groups. Data are expressed as mean ± SEM of at least 5 independent experiments.

The spindle abnormalities have been widely reported in aging MII oocytes. Owing to oxidative stress’s negative effects on meiotic spindle integrity, we determined whether reduced oxidative stress in aging MII oocyte induced by resveratrol treatment could facilitate maintenance of spindle morphology. Immunofluorescent results revealed that more than half of the oocytes in control and resveratrol-treated group exhibited normal spindle with a typical barrel-shape positioned beneath the cortex (Figure 6A). Meanwhile, abnormal spindles were also observed in both groups. Morphological abnormalities of spindles in this experiment mainly included elongated spindles, spindles without poles, mono-polar spindles, multi-poles and unorganized spindles with astral microtubules as well as many cytoplasmic asters (Figure 6A). The proportions of defective spindles were similar in the resveratrol-treated group and the control group (60.53, 39.47% n= 120, vs. 61.54, 38.46%, n= 170, P > 0.05) (Figure 6B).

## DISCUSSION

When a woman reaches her mid-thirties, fertility becomes decreased, and the oocytes undergo a decline in developmental potential with increasing maternal age. Although the molecular mechanisms are complex, a convincing link between oxidative stress and age-related decline in oocyte quality is well established [39]. In addition, oxidative stress also affects the quality of postovulatory oocytes both *in vivo* and *in vitro* processes [10,40]. Accordingly, it has become particularly pressing to find an effective treatment to prevent or delay the damage caused by oxidative stress. As a promising anti-oxidative small molecule, the effects of resveratrol on preventing chemical-induced oxidative damage or apoptosis have been extensively studied [32,33,41,42]. Furthermore, the possible functions of resveratrol in improving oocyte maturation, fertilization and preimplantation embryonic development have also been reported [36–38]. In addition, previous studies demonstrated that long-term treatment (12 months) of resveratrol was able to protect against age-associated infertility, as evidenced by an elevated follicle pool, decreased spindle aberrations and chromosome misalignments [28]. However, most of these studies were carried out *in vitro* and did not demonstrate the effects on delaying postovulatory oocyte aging. The present study fills this gap and provides important evidence for *in vivo* roles of resveratrol in delaying postovulatory oocyte aging.

In this study, we investigated the potential roles of resveratrol in delaying postovulatory oocyte aging *in vivo*. The 7-8 month-old female mice were selected as experimental subjects because the fertility begins to decrease from that age on. Our data showed that short-term injection of resveratrol delayed aging and apoptosis of postovulatory oocytes, which were possibly achieved by attenuating oxidative stress and preserving mitochondrial function. Our results showed that resveratrol treatment significantly decreased ROS levels and elevated the expression of SIRT1 in postovulatory oocytes, which was consistent with previous *in vitro* studies [32,43]. Oxidative stress is related to age-related mitochondrial dysfunction. Further studies indicated that resveratrol-treatment did not impact mitochondrial distribution but significantly decreased mitochondrial copy number in aging oocytes. One possible reason was that resveratrol treatment protected mitochondrial DNA from impairment by oxidative stress in aging oocytes and improved mitochondrial function, which was consistent with previous studies in patient-derived fibroblasts [44] and porcine oocytes [36]; accordingly, it was not necessary to increase mitochondria copy number to compensate mitochondrial dysfunction in resveratrol treatment group compared to control group.

Previous studies showed that chronic administration of resveratrol (6 months) prevented age-associated spindle abnormalities [28]. However, our experiment results found that short-term resveratrol treatment did not prevent spindle aberrations in aging oocytes. It is probably that low dosage and short-term treatment of resveratrol may not be sufficient to rescu the spindle aberrations in aging oocytes. Besides, similar results were observed in study of another antioxidant N-Acetyl-L-cysteine (NAC), in which NAC-treatment for 6 months showed no obvious improvement on spindle aberrations [15]. Another *in vitro* study found that the short-term treatment of Nicotinamide (NAM), a SIRT1, 2, 3 inhibitor, significantly elevated ROS levels but did not aggravate spindle abnormalities in aging oocytes [13].

In summary, *in vivo* administration of resveratrol decreases fragmentation and death of oocyte aging in the oviduct, probably by improving mitochondrial function, increasing SIRT1 expression and preventing ROS production. The results of this research may provide reproductive health care providers with new clues to improve their clinical practice. Administration of resveratrol may be an effective treatment to improve the oocyte developmental potential and increase the probability of getting pregnancy either naturally or through assisted reproductive technique. However, the effectiveness and safety of resveratrol require verification by further studies involving human subject.

## MATERIALS AND METHODS

### Mice

Female ICR mice were housed in a temperature-controlled room with 12D:12L (dark vs. light). Water and food were available ad libitum. All animal operations conformed to the guidelines by the Animal Research Committee principles of the Institute of Zoology, Chinese Academy of Sciences. A stock solution of resveratrol was diluted in absolute ethanol to 50g/L and stored at -20°C. Prior to administration, the stock solution was diluted with normal saline (0.9% NaCl) to a final concentration of 2.5g/L. Female ICR mice (aged 7-8 months) were randomly divided into experiment groups and control groups. For the experiment groups, resveratrol (Puhuashi Technology Development (Beijing) Co., Ltd, China; HPLC ≥ 99%) was administrated by intraperitoneal injection (50 mg/kg BW/day) for 15 consecutive days (Table 1). Control groups received injection of normal saline with 5% of ethanol (50 mg/kg BW/day) for the same period (Table 1). Body weights were assessed every 3 days during the experiment and injection doses were adjusted accordingly.

**Table 1:** A time table for the treatment of animals and sample collection.

	**Day 1-12**	**Day 13**	**Day 14**	**Day 15**	**Day 16**
9:00		PMSG		hCG	Egg Retrieval
19:00	Res/5% Ethanol	Res/5% Ethanol	Res/5% Ethanol	Res/5% Ethanol	

### Tissue and oocyte collection

To collect MII eggs, all mice were injected with PMSG (10 IU) in the thirteenth day of injection, 48 h later with hCG (10 IU) (Table 1). Prior to oocyte collection, mice were weighted and sacrificed by cervical dislocation. Eggs were retrieved from oviductal ampullae 24 h after hCG (Table 1), and cumulus cells were removed by gentle pipetting in M2 (Sigma, USA) medium containing 0.3% hyaluronidase. Then denuded oocytes were examined, or collected and stored in -80°C. Ovaries were weighted and placed in 4% paraformaldehyde before subsequent processing.

### Hematoxylin and eosin staining

Ovaries were fixed in 4% paraformaldehyde overnight at 4°C, dehydrated with a graded ethanol series and embedded in paraffin. Paraffin-embedded ovaries were cut into sections of 8-μm thickness and mounted on glass slides. After adequately drying for 45 °C overnight, sections were deparaffinized in xylene, hydrated by a graded alcohol series and stained with hematoxylin and eosin for histological analyses.

### Western blotting

For western blotting, 120 oocytes were lysed in 2×SDS sample buffer, boiled for 5 min at 100°C and then subjected to 10% sodium dodecyl sulfate polyacrylamide gel electrophoresis (SDS-PAGE). The separated proteins were transferred to a polyvinylidene difluoride (PVDF) membrane. Membrane was blocked in Tris-buffered saline (TBS) containing 5% BSA and 0.1% Tween-20 for 2 h and then incubated with rabbit anti-SIRT1 antibody (1:1000) (9475; Cell Signaling Technology, Inc.) and mouse anti-β-actin antibody (1:1000) (BE0021; Easybio Technology, Beijing). After multiple washes in TBS containing 0.1% Tween-20 and incubation with horseradish peroxidase (HRP)-conjugated anti-rabbit IgG (1:1000) (ZB-2301; Zhongshan Golden Bridge Biotechnology, Beijing) and horseradish peroxidase conjugated anti-mouse IgG (1:1000) (ZB-2305; Zhongshan Golden Bridge Biotechnology, Beijing), respectively, finally, the membranes were washed 3 times in TBS containing 0.1% Tween-20 and visualized using Bio-Rad ChemiDoc XRS+.

### Quantitative RT-PCR analysis

Total RNA was extracted from 50 oocytes using RNeasy Micro Kit (Qiagen). The first strand cDNA was generated with cDNA synthesis kit (Invitrogen). *Sirt1* genes were amplified. *Glyceraldehyde-3-phosphate dehydrogenase (Gapdh)* was used as control gene to correct cDNA levels of samples. Real-time PCR was conducted by using UltraSYBR Mixiure (CoWin Biosciences (Beijing) Co., Ltd., China) in Roche LightCycler480 II detection system. The relative gene expression was calculated by the 2^-ΔΔCt^ method. The primers used were as follows. *Sirt1* forward: 5′-TATCTATGCTCGCCTTGCGG-3′; *Sirt1* reverse 5′-CGGGATATATTTCCTTTGCAAACTT-3. *Gapdh* forward: 5′-CCCCAATGTGTCCGTCGTG-3′; *Gapdh* reverse 5′-TGCCTGCTTCACCACCTTCT-3.

### Immunofluorescence

Immunofluorescence was performed by using previously published methods [45]. Briefly, oocytes were fixed for 30 min at room temperature in 2% formaldehyde supplemented with 100mM HEPES, 50mM EGTA, 10mM MgSO_4_, 0.2% Triton X-100 (pH=7, titrated with KOH). Then they were treated with PBS, 0.1% Triton X-100 overnight at 4^o^C and incubated with anti-α-tubulin-FITC antibody (F2168, Sigma, USA) (1:1000 in PBS with 0.1% Triton X-100 and 3% BSA) overnight at 4^o^C. Chromosomes were stained with DAPI for 15 min. The oocytes were mounted on glass slides and examined with a laser scanning confocal microscope (Zeiss 780 META).

For staining active mitochondria, eggs were incubated for 30 min at 37°C in M2 medium containing 200nM MitoTracker ® Red CMXRos (M7512, Invitrogen, USA). After washing 3 times, eggs were stained with Hoechst 33342 (10mg/ml) for 15 min. Finally, eggs were mounted on glass slides and examined with a Perkin Elmer UltraVIEW VOX confocal Imaging System.

### Detection of intracellular ROS levels

ROS levels detection was performed as previously reported [13]. Briefly, oocytes were incubated for 30 min at 37°C in M2 medium supplemented with 2',7'-dichlorodihydrofluorescein diacetate (DCFA-DA) (2 μM) (Beyotime Institute of Biotechnology (Shanghai) Co., Ltd., China). After washing three times in M2 medium, oocytes were stained with Hoechst 33342 (10mg/ml) for 15 min. Finally, oocytes were mounted on glass slides and examined in identical settings with Perkin Elmer UltraVIEW VOX confocal Imaging System.

### Detection of mtDNA copy number

MtDNA copy number in oocytes was assessed according to the method previously described [46]. mtDNA-specific primers: B6 forward, 5′-AACCTGGCACTGAGTCACCA-3′, and B6 reverse, 5′-GGGTCTGAGTGTATATATCATGAAGAGAAT-3′ were used to prepare a standard amplification curve for absolute quantification of mtDNA. PCR products were ligated to the T-vector and amplified in E. coli. Then, plasmids were extracted and purified, followed by 10-fold serial dilutions for seven times. Standard curve was established afterwards. One oocyte was placed into a PCR tube with 5 µl lysis buffer and incubated at 55°C for 2 h. After heat inactivation of proteinase K at 95°C for 10 min, lysates were used for real-time PCR using Roche LightCycler480 II detection system and the above-mentioned B6 primers. Reaction conditions of PCR were as follows: initiation at 95°C for 10 min, 40 cycles of 95°C treatment for 10 s and 60°C reaction for 1 min. The melting temperature was set at 76.5°C. All standard curves of these samples were processed by linear regression, which showed a correlation coefficient of 0.98. All measurements were repeated three times.

### Statistical analysis

All experiments were performed at least three times. Data were presented as mean ± SEM. Data were evaluated by Student’s t-test. *P*<0.05 was considered statistically significant.
